# Exploring the potential of oxymatrine in preventing CHIKV-induced acute kidney injury based on multi-dimensional computational analysis and *in vitro* experiments

**DOI:** 10.3389/fmicb.2026.1782183

**Published:** 2026-04-02

**Authors:** JiaLiang Xin, FangJun Li, LuLu Kang, Jie Yang, HaiTong Wang, Wei Wang, Sheng Feng, XinFei Liao, WenJie Li, He Zhang

**Affiliations:** 1 Jilin Collaborative Innovation Center for Antibody Engineering, Jilin Medical University, Jilin, China; 2 Key Laboratory of Animal Disease and Human Health of Sichuan Province, College of Veterinary Medicine, Sichuan Agricultural University, Chengdu, China; 3 Guangxi Key Laboratory of Animal Breeding, Disease Control and Prevention, College of Animal Science and Technology, Guangxi University, Nanning, China; 4 Wenzhou Municipal Center for Disease Control and Prevention, Wenzhou Municipal Institute of Health Supervision, Wenzhou, China; 5 Wenzhou Polytechnic, Wenzhou, China; 6 Changchun Veterinary Research Institute, Chinese Academy of Agricultural Sciences, Changchun, China

**Keywords:** acute kidney injury, CHIKV, ferroptosis, network pharmacology, Oxymatrine

## Abstract

Chikungunya virus (CHIKV) infection can induce acute kidney injury (AKI) and may be fatal in severe cases, yet effective therapeutic strategies remain unclear. This study integrated computational analysis (network pharmacology and molecular docking) with *in vitro* experiments to evaluate the protective potential of oxymatrine against CHIKV-induced AKI and to explore its underlying mechanisms. Network pharmacology analysis identified 605 overlapping targets between CHIKV- and AKI-related genes, with core targets including TNF, AKT1, IL6, IL1B, and TP53, which were primarily enriched in the PI3K/AKT signaling pathway. RT-qPCR analysis and molecular docking further indicated that oxymatrine may interact with 18 targets, such as BAX, BCL2, and TLR4, thereby modulating CHIKV-associated PI3K/AKT signaling. *In vitro* experiments showed that oxymatrine at concentrations of 250–2,000 μM significantly increased the viability of HEK293T cells infected with CHIKV at a MOI of 0.01 for 24 h, with an average increase of approximately 25.8%. At these concentrations, oxymatrine reduced the expression of the CHIKV entry factor *TIM-1* and regulated the PI3K/AKT, NF-κB, and TNF signaling pathways, which may contribute to the inhibition of viral replication. Further analysis revealed that CHIKV-induced AKI involved 75 ferroptosis-related targets and was closely associated with inflammatory responses. Oxymatrine may attenuate these effects through the regulation of targets such as SIRT3, AR, and FURIN. Consistently, ELISA results demonstrated that 1,000 μM oxymatrine significantly decreased the levels of IL-1β, TNF-α, and IL-6 in HEK293T cells infected with CHIKV. In conclusion, these findings suggest that oxymatrine may protect against CHIKV-induced AKI by limiting viral replication, modulating PI3K/AKT and NF-κB/TNF signaling pathways, suppressing inflammation, and regulating ferroptosis-related processes.

## 1 Introduction

Chikungunya virus (CHIKV) is a mosquito-borne, single-stranded, positive-sense RNA virus classified within the genus *Alphavirus* of the family *Togaviridae* (Rougeron et al., 2015). Since the early 2000s, CHIKV has caused repeated large-scale outbreaks in Africa, Asia, and the Americas, and it is now recognized as a growing global public health concern (Vega-Rúa et al., 2020; Staples et al., 2009). Infection with CHIKV is typically characterized by acute fever, severe joint pain, and pronounced inflammatory responses (Thiberville et al., 2013). In recent years, however, increasing clinical and experimental evidence has shown that CHIKV infection is not limited to the musculoskeletal system. Instead, it can lead to damage in multiple organs, including the nervous system, cardiovascular system, and kidneys (Traverse et al., 2022; Cheng et al., 2025; Mercado et al., 2018). Among these complications, acute kidney injury (AKI) is particularly severe. In hospitalized patients with CHIKV infection, the reported incidence of AKI varies considerably across studies, ranging from approximately 21% to 45%, and is predominantly associated with severe or atypical clinical presentations (Elahi et al., 2025). The development of AKI is closely linked to rapid disease progression and a significantly increased risk of mortality in patients with CHIKV infection (Mercado et al., 2018).

Despite these observations, the mechanisms responsible for CHIKV-induced renal injury have not yet been fully elucidated. Available evidence has suggested that kidney damage results from the combined effects of several pathological processes. One important factor is the excessive immune and inflammatory response triggered by CHIKV infection, which leads to the release of large amounts of pro-inflammatory cytokines and chemokines. This response can directly impair both glomerular and tubular structures (Mercado et al., 2018; Elahi et al., 2025; Sánchez-Arcila et al., 2020; Venugopalan et al., 2014). At the same time, virus-induced oxidative stress, mitochondrial dysfunction, and activation of apoptosis-related pathways have been reported to contribute to renal cell injury (Dhanwani et al., 2012). In addition, endothelial dysfunction and disturbances in renal microcirculation may further worsen ischemia and hypoxia in kidney tissue, ultimately accelerating the decline in renal function (Joubert et al., 2012). Our previous work also demonstrated that CHIKV-induced kidney injury involves multiple signaling pathways, including the PI3K/Akt pathway, ferroptosis, and apoptosis (Cheng et al., 2025). Notably, activation of the PI3K/Akt pathway has been shown to support intracellular replication of CHIKV, suggesting its dual role in both viral propagation and tissue injury (Thaa et al., 2015).

Although these studies have improved our understanding of CHIKV-associated AKI, effective therapeutic options remain very limited. At present, no specific antiviral drugs are available for CHIKV infection, and clinical treatment mainly relies on supportive care. This limitation is particularly evident in patients with renal complications, for whom targeted treatment strategies are lacking. Under these circumstances, there is a clear need to explore potential agents that combine anti-inflammatory, antioxidant, and renoprotective effects.

Oxymatrine is a quinazoline alkaloid extracted from the traditional Chinese medicinal herb *Sophora flavescens* and has received increasing attention in recent years. Previous studies have shown that Oxymatrine can reduce hepatocyte apoptosis in a rat model of acute liver failure induced by lipopolysaccharide and D-galactosamine, mainly through regulation of the TLR4/PI3K/Akt/GSK-3β signaling pathway (Zhang et al., 2017). Oxymatrine has also been reported to alleviate renal ischemia-reperfusion injury by activating the Nrf2/HO-1 pathway (Jiang et al., 2015). In addition, several studies have indicated that Oxymatrine can modulate ferroptosis under different pathological conditions (Gao et al., 2024; Zeng et al., 2025). Although its direct antiviral activity against CHIKV has not yet been confirmed, these pharmacological properties are closely related to the key mechanisms involved in CHIKV-induced renal injury.

Based on this background, we hypothesized that Oxymatrine might protect protect against AKI caused by CHIKV infection through coordinated regulation of multiple targets and pathways. In this study, we combined computational analysis (network pharmacology analysis and molecular docking) with *in vitro* experiments to explore the potential effects and underlying mechanisms of Oxymatrine in CHIKV-induced AKI. The results are expected to provide experimental evidence and a theoretical basis for further evaluation of Oxymatrine as a potential therapeutic option for CHIKV-induced AKI.

## 2 Materials and methods

### 2.1 Cell lines, viruses, and compounds

HEK293T and BHK-21 cells (China Center for Type Culture Collection) were cultured in DMEM (Gibco) supplemented with 10% FBS (Gibco), 100 U/mL penicillin, and 100 μg/mL streptomycin (Thermo Fisher Scientific) at 37 °C with 5% CO_2_.

The Chikungunya virus 0706a TW (GenBank accession no. EU703760.1) was provided by the Changchun Veterinary Research Institute and stored at −80 °C. Oxymatrine (MedChemExpress, HY-N0158, purity ≥99.9%) was dissolved in sterile distilled water as a 100 mM stock solution.

### 2.2 Computational prediction

#### 2.2.1 Acquisition of disease- and compound-related targets

CHIKV-related targets were identified following a previously reported approach (Xin et al., 2025). Acute kidney injury (AKI)-related targets were collected by searching the keywords “acute kidney injury” in MalaCards (https://www.malacards.org), GeneCards (https://www.genecards.org), and Online Mendelian Inheritance in Man (OMIM, https://www.omim.org). Each database was queried separately to maximize coverage and reliability.

Targets associated with oxymatrine were retrieved from the Traditional Chinese Medicine Systems Pharmacology database (TCMSP, https://old.tcmsp-e.com/tcmsp.php), SwissTargetPrediction (http://www.swisstargetprediction.ch/), HERB (https://herb.ac.cn), and STITCH (https://stitch-db.org). Duplicates were removed, and a non-redundant target set was generated. Venn analysis was used to identify targets overlapping between CHIKV-induced AKI and oxymatrine-related targets, representing potential therapeutic targets.

#### 2.2.2 Protein-protein interaction network construction and analysis

Potential key targets were analyzed using the STRING database. To minimize network fragmentation, the PPI network was constructed using a confidence score threshold of 0.4 and restricted to *Homo sapiens*. The network was imported into Cytoscape (version 3.9.1), and the CytoNCA plugin was used to assess topological features with Degree, Betweenness, and Closeness measures.

To explore the underlying pathogenic mechanisms, the MCODE plugin was applied to the PPI network. Subclusters containing at least 10 nodes were identified using a K-Core parameter of 2 and visualized for further analysis.

#### 2.2.3 KEGG pathway analysis

Core pathways involved in CHIKV-induced AKI and those potentially modulated by oxymatrine were identified using the DAVID database (https://david.ncifcrf.gov/). Target sets from the three main disease-related subclusters and the oxymatrine-related therapeutic targets were analyzed separately. Kyoto Encyclopedia of Genes and Genomes (KEGG) pathway enrichment analysis was performed, and the eight pathways with the lowest *p*-values from each set were selected for visualization.

### 2.3 Molecular docking validation

Three-dimensional structures of the target proteins were retrieved from the Protein Data Bank (PDB, https://www.rcsb.org), focusing on human proteins determined by X-ray crystallography with a resolution below 3 Å. Only structures corresponding to the top 10 PPI targets were considered.

The two-dimensional structure of oxymatrine was obtained from PubChem (https://pubchem.ncbi.nlm.nih.gov/). Both protein and ligand structures were imported into CB-DOCK2 (http://cadd.labshare.cn/cb-dock2/php/index.php) for blind docking. Docking complexes were visualized, with attention given to those exhibiting the lowest Vina scores.

### 2.4 *In vitro* experiment verification

#### 2.4.1 Safe dose determination of the compound

HEK 293T cells were seeded in 96-well plates and cultured for 24 h. Cells were then treated with varying concentrations of oxymatrine in DMEM containing 2% FBS, while a control group received only the solvent. After 48 h, cell viability was assessed using the CCK-8 assay (Vazyme, A311-01, China). Each experiment was repeated six times.

#### 2.4.2 Assessment of compound protection against CHIKV infection

To evaluate the antiviral effect of oxymatrine, HEK293T cells were seeded in 96-well plates and pretreated with different concentrations of the compound for 2 h. Cells were then infected with CHIKV at a MOI of 0.01 for 2 h. Fresh medium containing the corresponding oxymatrine concentrations was added, and cells were incubated for 48 h before viability assessment using the CCK-8 assay.

For further analysis, cells were cultured in 12-well plates and treated similarly. After infection, supernatants and cells were collected separately at 24 h. Cellular RNA was extracted to measure CHIKV Nsp2 gene expression and E1 protein levels. Relative RNA abundance was calculated using the 2^−ΔΔ*CT*^ method (primer sequences listed in Supplementary material 1). Supernatants were transferred to BHK-21 cells in 12-well plates (100 μL per well). After 2 h, the supernatant was replaced with DMEM containing 2% FBS. Cell viability was evaluated by crystal violet staining after 48 h.

#### 2.4.3 Evaluation of the protective effect of compound addition time on cells infected with CHIKV

HEK293T cells were cultured in 96-well and 12-well plates and infected with CHIKV at an MOI of 0.01 for 2 h. Oxymatrine was added either 2 h before or 2 h after infection for a treatment duration of 2 h. Cell viability in 96-well plates was assessed at 24 h using the CCK-8 assay. Cells from 12-well plates were collected for RNA extraction to measure CHIKV Nsp2 gene expression, and relative RNA levels were quantified using the 2^−ΔΔ*CT*^ method (primer sequences listed in Supplementary material 1).

### 2.5 Investigation on the *in vitro* Inhibition of CHIKV by oxymatrine

#### 2.5.1 The influence of oxymatrine on the CHIKV entry factor

To assess whether oxymatrine could suppress cytokine-related responses and interfere with CHIKV adsorption, HEK293T cells were seeded into 12-well plates. After 24 h of incubation, the culture medium was removed. Oxymatrine, prepared in DMEM containing 2% FBS, was added to each well at a volume of 100 μL and the cells were incubated for 12 h. The cells were then infected with CHIKV at a MOI of 0.01 for 2 h. After infection, the inoculum was discarded, fresh culture medium was added, and the cells were incubated for an additional 24 h.

Cells were subsequently harvested, total RNA was extracted, and RT-qPCR was performed to quantify the expression of *ACTG1, FHL1, TIM-1, COL1A2, PTPN2*, and *IFITM3*. Three control groups were included: an oxymatrine-only group, a healthy control group treated with vehicle alone, and a virus infection group treated with vehicle following CHIKV exposure. Primer sequences used for RT-qPCR are provided in Supplementary Table 1. The molecular docking procedure described previously was also applied to support and verify the experimental findings.

#### 2.5.2 The effect of oxymatrine on CHIKV-related pathogenic targets

To clarify how oxymatrine influences intracellular CHIKV replication, HEK293T cells were infected with CHIKV at a MOI of 0.01 for 2 h and then treated with oxymatrine. Total RNA was collected 24 h after infection. The expression of several key genes, including *CASP3, MMP9, BAX, CTSB, TLR4*, and *TLR2*, was measured by RT-qPCR. Relative RNA levels were calculated using the 2^−ΔΔ*Ct*^ method. Primer sequences are provided in Supplementary Table 1.

To further explore whether oxymatrine is associated with the NF-κB, TNF, and PI3K/AKT signaling pathways, molecular docking analysis was also performed. The binding affinities between oxymatrine and AKT1, NFKB1, TNF, RELA, and SRC were analyzed to support the experimental results.

#### 2.5.3 Evaluation of the ferroptosis effect of oxymatrine on CHIKV

To examine whether oxymatrine is involved in ferroptosis during CHIKV-induced acute kidney injury, ferroptosis-related targets were obtained from the FerrDB database. The overlap between CHIKV-associated AKI targets and ferroptosis-related genes was analyzed using Venn analysis. Targets related to oxymatrine were also included to identify potential shared genes involved in this process.

Cytoscape version 3.9.1 was used to construct and analyze the interaction network, allowing the identification of core targets within the ferroptosis-related gene set associated with CHIKV-induced AKI. Venn analysis was further applied to evaluate the involvement of oxymatrine in the top 20 core targets. Molecular docking was performed to validate the interactions between oxymatrine and these candidate targets.

In addition, ELISA was used to measure the levels of TNF, IL-6, and IL-1β in the culture supernatant. HEK293T cells were seeded in 6-well plates, infected with CHIKV at a MOI of 0.01, and subsequently treated with oxymatrine. Cytokine concentrations were determined according to the manufacturer's instructions.

### 2.6 Statistical analysis

The experimental data were statistically analyzed using GraphPad Prism 5.0. Significance levels were determined using the unpaired Student's *t*-test for two-group comparisons or one-way analysis of variance (ANOVA) for multiple group comparisons (*P* < 0.05 denoted statistical significance).

## 3 Results

### 3.1 CHIKV induces AKI through multiple pathways

**Figure 1:**
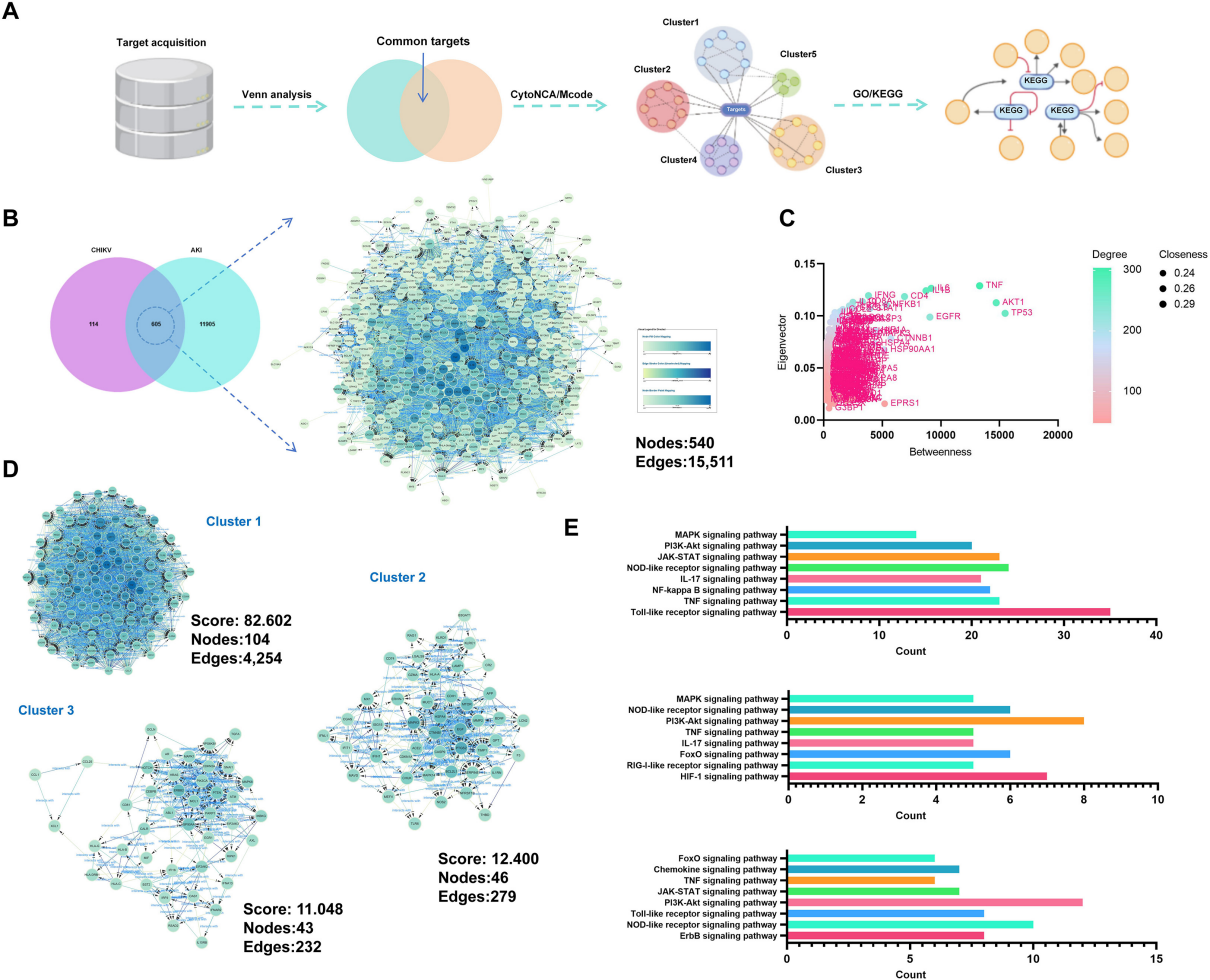
Identification of pathways involved in CHIKV-induced acute kidney injury (AKI). **(A)** Workflow for identifying pathways in CHIKV-induced AKI. **(B)** Venn diagram showing the overlap between CHIKV-related targets (*n* = 719) and AKI-associated targets (*n* = 12,510), identifying 605 shared targets. **(C)** Core targets screened from the PPI network using four topological algorithms. **(D)** MCODE analysis of the PPI network, identifying three major subclusters. **(E)** KEGG pathway enrichment analysis of targets in each subcluster.

Figure 1A outlines the workflow used to identify pathways involved in CHIKV-induced acute kidney injury. Based on online databases, 719 CHIKV-related targets and 12,510 AKI-associated targets were collected. Venn analysis showed that 605 targets overlapped between the two datasets, as illustrated in Figure 1B. These shared targets were then imported into the STRING database to construct a protein-protein interaction network.

Four topological algorithms were applied to the network to screen for core targets, and the results are summarized in Figure 1C. TNF, AKT1, TP53, IL1B, and IL6 emerged as key nodes within the CHIKV-induced AKI network. To further clarify the main pathogenic pathways, MCODE analysis was performed, and clusters with scores of 10 or higher were selected. The identified clusters are shown in Figure 1D. Three major subclusters were observed in the PPI network. Because core targets such as TNF, AKT1, and TP53 were mainly concentrated in cluster 1, this cluster was considered the primary pathogenic module related to CHIKV-induced AKI.

KEGG pathway enrichment analysis was then conducted for targets in each subcluster. As shown in Figure 1E, the PI3K/AKT signaling pathway and the TNF signaling pathway were enriched in all three subclusters. In addition, pathways such as the MAPK signaling pathway and the JAK-STAT signaling pathway were also identified, indicating that they may participate in the development of CHIKV-induced AKI.

### 3.2 Oxymatrine is involved in multiple signaling pathways related to CHIKV-induced AKI

**Figure 2:**
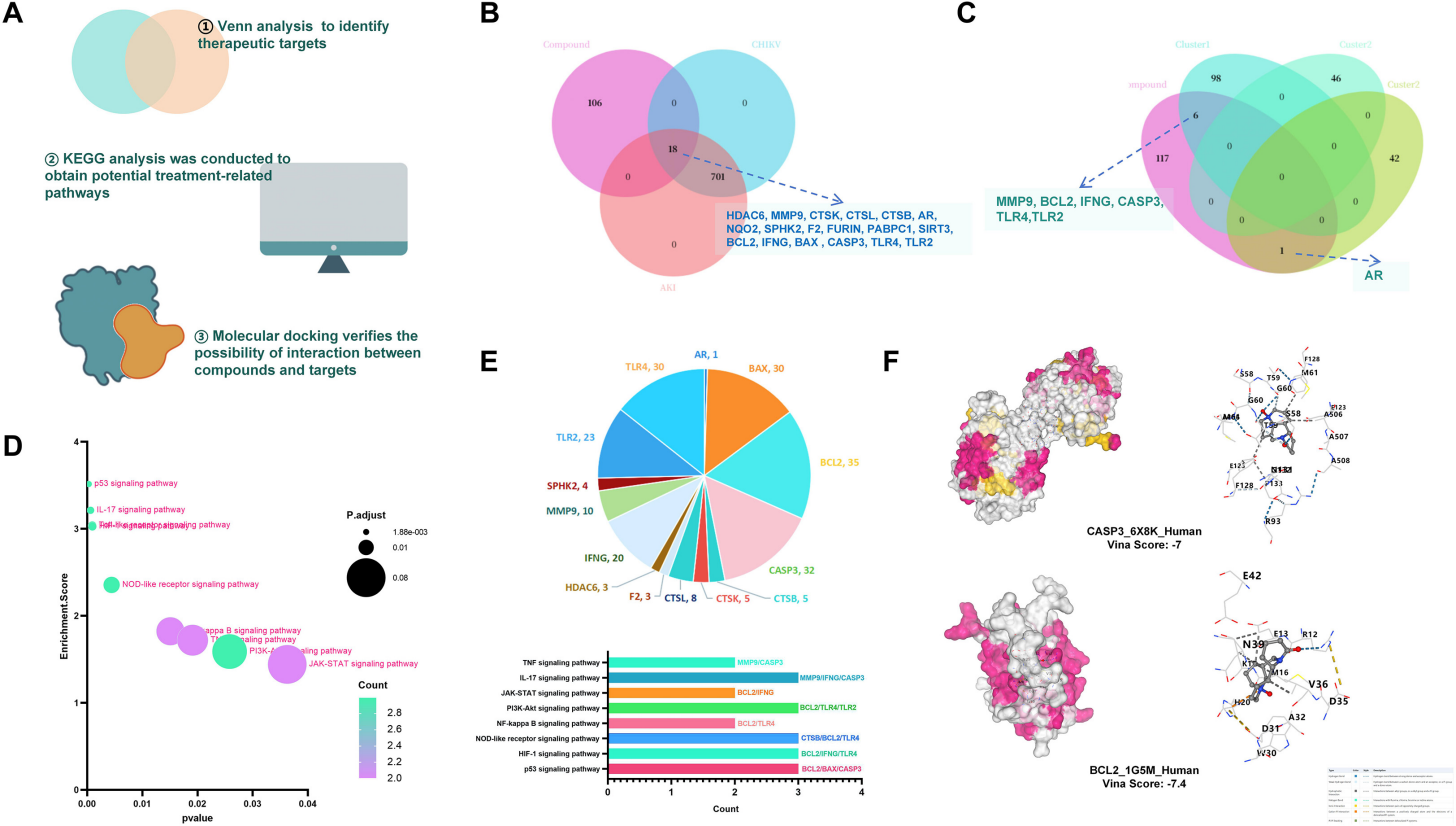
Network pharmacology analysis of oxymatrine in CHIKV-induced AKI. **(A)** Workflow for exploring the effects of oxymatrine on CHIKV-induced AKI. **(B)** Venn diagram identifying 18 overlapping targets between oxymatrine and CHIKV-induced AKI. **(C)** Intersection of oxymatrine targets with three subclusters of the disease. **(D)** KEGG pathway enrichment analysis of the 18 overlapping targets. **(E)** Representation of the most frequently involved targets across multiple enriched signaling pathways. **(F)** Binding details of oxymatrine to the target proteins CASP3 and BCL2.

Figure 2A presents the workflow of the network pharmacology analysis exploring the effects of oxymatrine on CHIKV-induced AKI. Venn analysis identified 18 overlapping targets between oxymatrine and CHIKV-induced AKI. These targets included BCL2, IFNG, BAX, CASP3, and TLR4, as shown in Figure 2B. Most of the overlapping targets were mainly distributed in subcluster 1 of the CHIKV-induced AKI network, as illustrated in Figure 2C.

KEGG pathway enrichment analysis showed that these 18 targets were involved in several signaling pathways. The most prominent pathways were the PI3K/AKT signaling pathway, the JAK-STAT signaling pathway, and the TNF signaling pathway, as shown in Figure 2D. Among the enriched pathways, BAX, BCL2, and CASP3 were the most frequently involved targets and appeared in multiple key signaling processes, as shown in Figure 2E.

Molecular docking analysis further supported the network pharmacology results. Oxymatrine was able to bind stably to human CASP3 and BCL2 proteins, with Vina scores lower than −7, indicating a relatively strong binding affinity (Figure 2F).

### 3.3 Oxymatrine inhibits CHIKV replication in HEK293T cells

**Figure 3:**
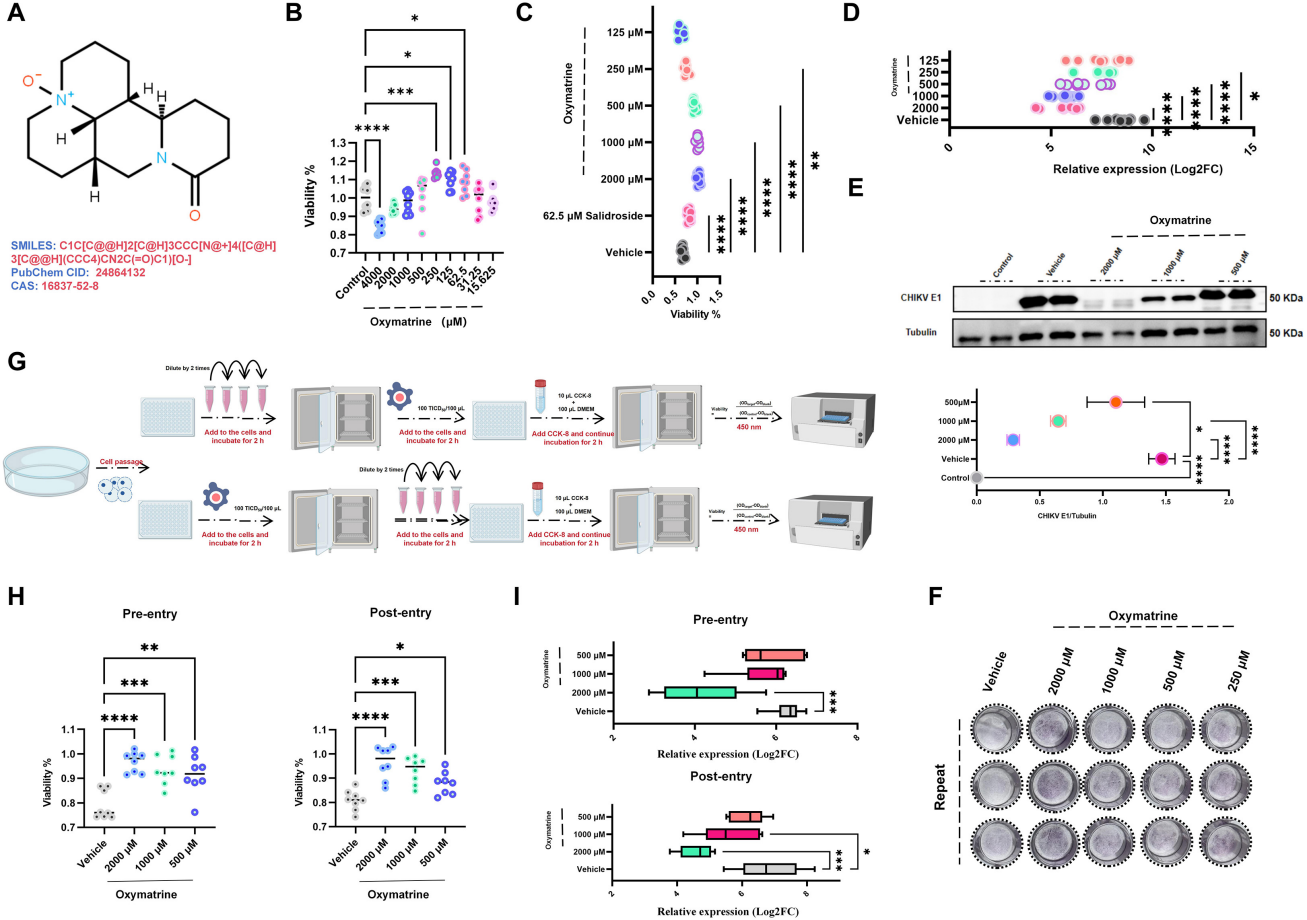
Oxymatrine inhibits CHIKV replication in HEK293T cells. **(A)** Chemical structure of oxymatrine. **(B)** Cell viability of HEK293T cells treated with oxymatrine at concentrations ranging from 15.625 to 2000 μM (*n* = 8). **(C)** Dose-dependent protective effect of oxymatrine (250–2,000 μM) on HEK293T cells infected with CHIKV (0.01 MOI) (*n* = 8). **(D)** RT-qPCR analysis showing oxymatrine-mediated reduction of CHIKV replication in infected cells (*n* = 6). **(E)** Western blot analysis of CHIKV E1 protein levels in infected cells treated with oxymatrine (500–2,000 μM) (*n* = 3). **(F)** Crystal violet staining showing increased survival of CHIKV-infected cells after oxymatrine treatment (250–2,000 μM). **(G)** Workflow of pre- and post-infection oxymatrine treatment. **(H)** CCK-8 assay comparing cell viability improvement with oxymatrine treatment at both time points (*n* = 8). **(I)** Comparison of the inhibitory effect on CHIKV replication between pre- and post-infection oxymatrine treatment by RT-qPCR (*n* = 6). Compared with Vehicle. All values represent the mean ± SD. ****P < 0.0001, ***P < 0.001, **P < 0.01, *P < 0.05.

The chemical structure of oxymatrine is shown in Figure 3A. Oxymatrine at concentrations ranging from 2,000 μM to 15.625 μM did not show cytotoxic effects on HEK293T cells. Instead, it moderately promoted cell viability, showing an overall positive trend (*p* < 0.05), as shown in Figure 3B. Although oxymatrine at 250 μM to 125 μM produced the most pronounced increase in cell viability, this effect did not directly reflect its antiviral activity in subsequent experiments.

In antiviral experiments, oxymatrine at concentrations between 2,000 μM and 250 μM showed a clear dose-dependent protective effect on HEK293T cells infected with CHIKV at an MOI of 0.01, as shown in Figure 3C. Consistent with this observation, RT-qPCR analysis demonstrated that oxymatrine within this concentration range reduced CHIKV replication in HEK293T cells (*p* < 0.05), as shown in Figure 3D.

Western blot analysis further supported these findings. Treatment with oxymatrine at concentrations from 2,000 μM to 500 μM limited the CHIKV-induced reduction of E1 protein levels in infected HEK293T cells (*p* < 0.05), as shown in Figure 3E. Crystal violet staining produced similar results. Oxymatrine at concentrations between 2,000 μM and 250 μM increased the number of surviving cells following CHIKV infection, as shown in Figure 3F.

To further assess the stage at which oxymatrine acts during the CHIKV life cycle, the compound was added either before or after viral infection. The experimental design is shown in Figure 3G. CCK-8 assay results indicated that oxymatrine treatment at either time point improved cell viability compared with untreated infected cells (*p* < 0.05), as shown in Figure 3H. Furthermore, at concentrations ranging from 500 to 2,000 μM, oxymatrine significantly inhibited CHIKV proliferation regardless of the time of administration (*p* < 0.05), as demonstrated in Figure 3I.

### 3.4 Oxymatrine may inhibit CHIKV adsorption to HEK293T cells by suppressing *TIM-1* expression

**Figure 4:**
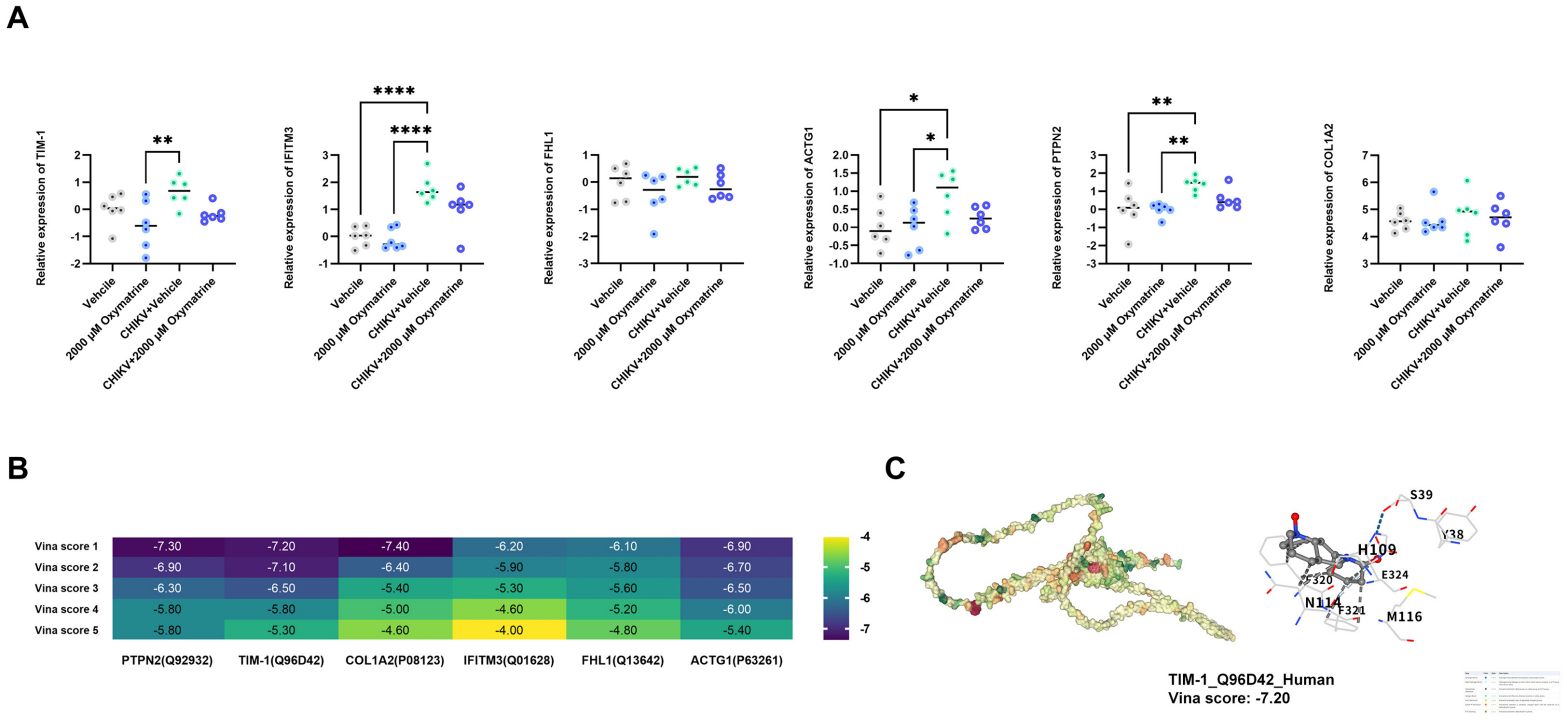
Oxymatrine may inhibit CHIKV adsorption by suppressing TIM-1 expression. **(A)** RT-qPCR analysis of six CHIKV adsorption-related factors in HEK293T cells, with or without CHIKV infection (*n* = 6). **(B)** Molecular docking analysis showing stable binding of oxymatrine to six adsorption-related proteins. **(C)** Detailed binding mode of oxymatrine with TIM-1 at the site with the lowest Vina score. All values represent the mean ± SD. ****P < 0.0001, ***P < 0.001, **P < 0.01, *P < 0.05.

To assess whether oxymatrine affects CHIKV adsorption to HEK293T cells, RT-qPCR was used to examine the expression of six known CHIKV adsorption-related factors in cells treated with oxymatrine, either in the presence or absence of viral infection. As shown in Figure 4A, treatment with 2,000 μM oxymatrine significantly downregulated *TIM-1* gene expression in both CHIKV-infected and uninfected HEK293T cells (*p* < 0.05). In contrast, the expression of the other adsorption-related factors showed no consistent changes.

Molecular docking analysis further supported these observations. Oxymatrine was able to form stable complexes with PTPN2, TIM-1, COL1A2, IFITM3, *FHL1*, and ACTG1, as shown in Figure 4B. Although the lowest Vina score for the TIM-1-oxymatrine complex was not the strongest among all tested targets, the average Vina score across its five binding sites was lower than those of the other complexes, suggesting relatively stable overall binding.

The detailed binding pattern of oxymatrine with TIM-1 at the site showing the lowest Vina score is presented in Figure 4C. These results suggest that oxymatrine may interfere with CHIKV adsorption by downregulating *TIM-1* expression and directly interacting with the TIM-1 protein.

### 3.5 Oxymatrine may reduce CHIKV-induced damage in HEK293T cells through the multi-gene targets

**Figure 5:**
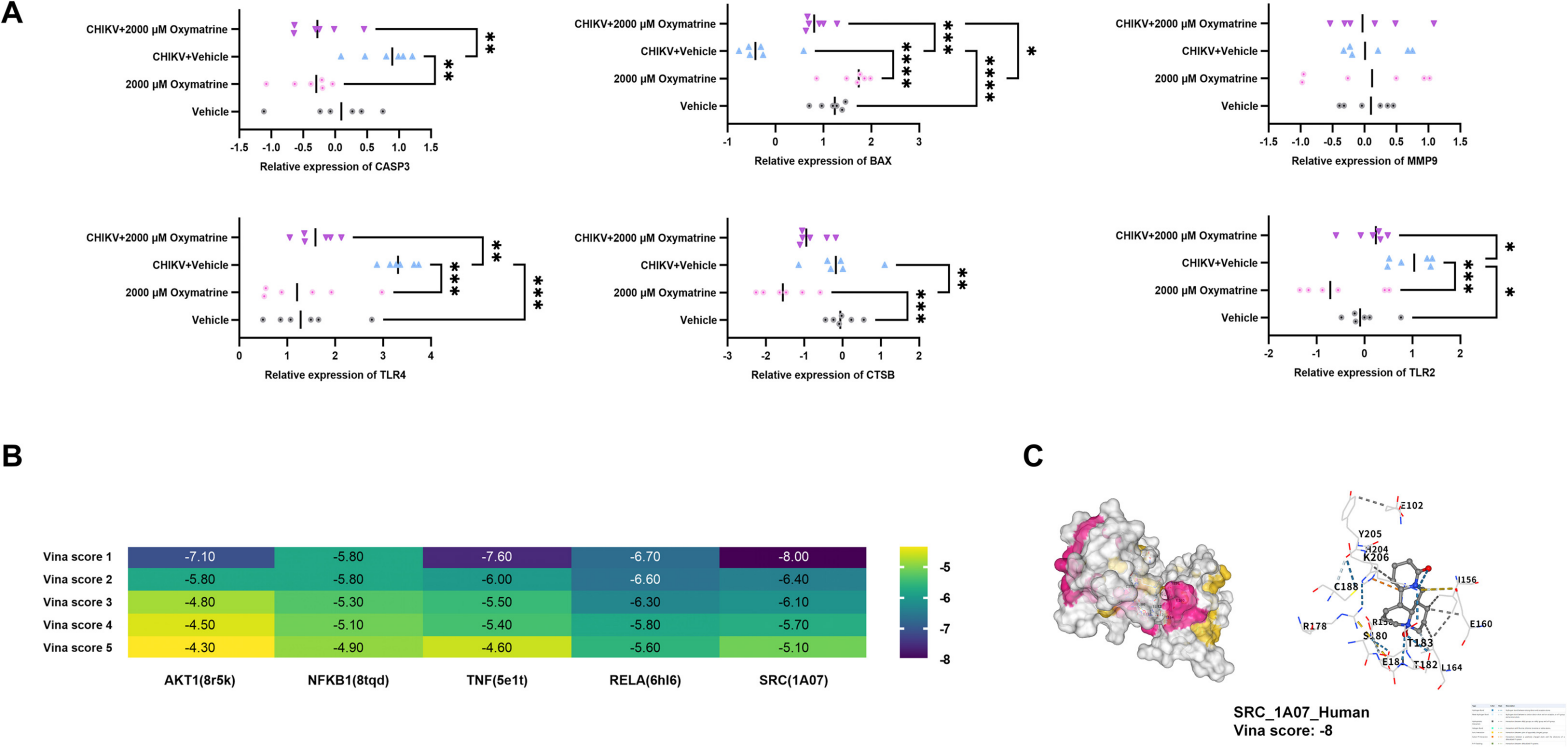
Oxymatrine may reduce CHIKV-induced damage through multi-gene targets. **(A)** RT-qPCR analysis of CHIKV-related gene expression in HEK293T cells, with or without infection, after oxymatrine treatment (*n* = 6). **(B)** Molecular docking analysis predicting the binding of oxymatrine to key signaling proteins. **(C)** Detailed binding mode of the oxymatrine-SRC complex (Vina score = −8). All values represent the mean ± SD. ****P < 0.0001, ***P < 0.001, **P < 0.01, *P < 0.05.

To explore the signaling pathways involved in the protective effects of oxymatrine against CHIKV in HEK293T cells, RT-qPCR was used to examine the expression of CHIKV-related genes in both infected and uninfected cells following oxymatrine treatment. The results are shown in Figure 5A. Oxymatrine significantly reduced the expression of *CASP3, TLR2, CTSB*, and *BAX* in HEK293T cells (*p* < 0.05), regardless of CHIKV infection status. These changes suggest that oxymatrine may attenuate virus-associated cellular responses by regulating genes linked to inflammation and apoptosis.

Molecular docking analysis provided further insight into the potential mechanisms involved. Oxymatrine was predicted to bind with several key signaling proteins, including AKT1, NFKB1, TNF, RELA, and SRC, as shown in Figure 5B. Among these interactions, binding to SRC was the most stable, with a Vina score of −8. The detailed binding mode of the oxymatrine-SRC complex is shown in Figure 5C. Together, these findings suggest that oxymatrine may reduce CHIKV-induced cellular damage by modulating the NFKB1-related signaling pathway.

### 3.6 Oxymatrine may intervene in ferroptosis in CHIKV-infected HEK293T cells

**Figure 6:**
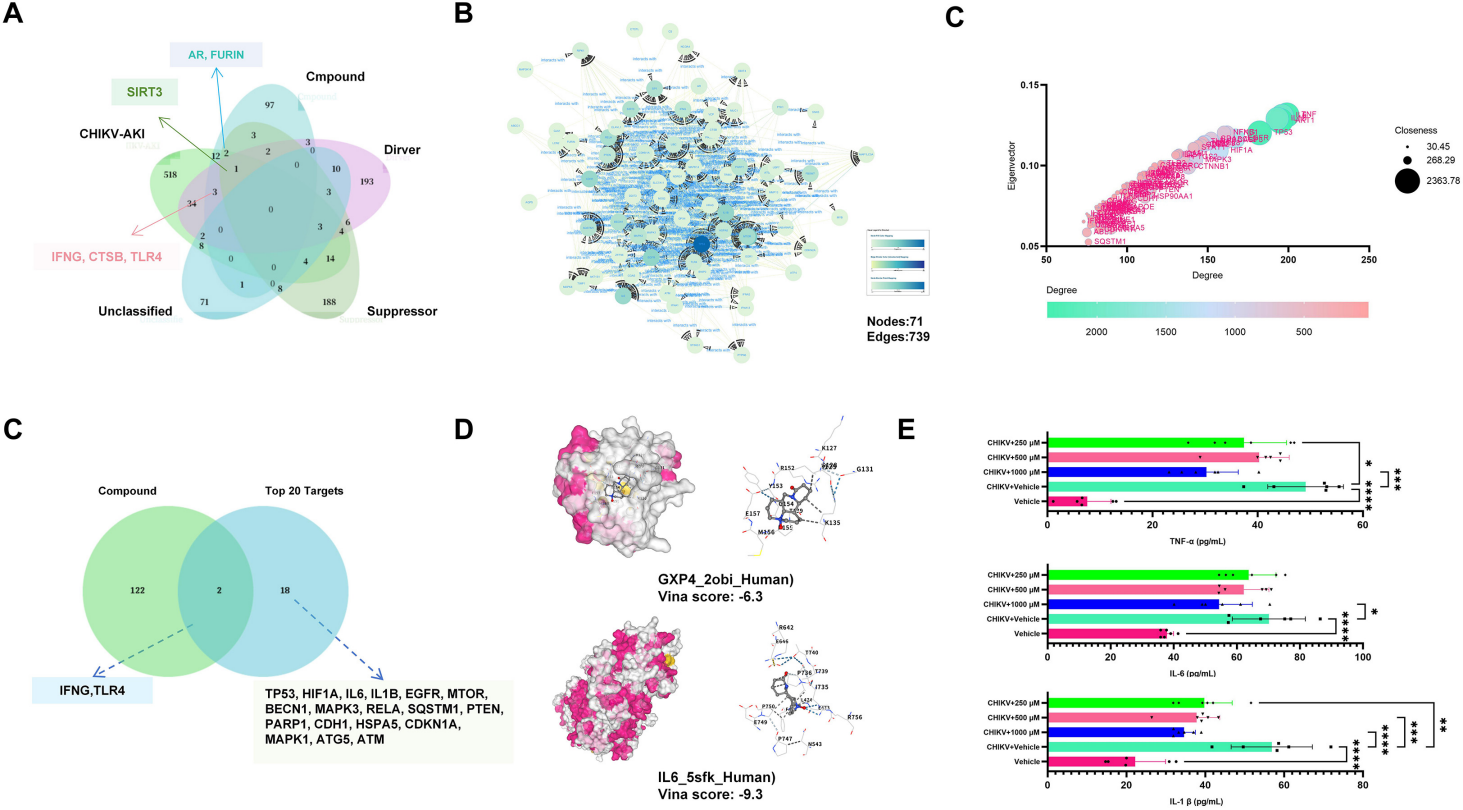
Oxymatrine may intervene in ferroptosis in CHIKV-infected HEK293T cells. **(A)** Venn diagram showing 75 ferroptosis-related targets in CHIKV-induced AKI, of which 7 are potential oxymatrine targets. **(B)** PPI network of the 75 ferroptosis-related targets. **(C)** Top 20 highly connected ferroptosis-related targets identified by topological analysis. **(D)** Molecular docking of oxymatrine with human GPX4 (Vina score = −6.3) and IL6 (Vina score = −9.3) proteins. **(E)** ELISA measurement of inflammatory cytokines (IL-1β, TNF-α, IL-6) in CHIKV-infected HEK293T cells treated with oxymatrine (500–1,000 μM) (*n* = 6). Compared with CHIKV + Vehicle. All values represent the mean ± SD. ****P < 0.0001, ***P < 0.001, **P < 0.01, *P < 0.05.

Venn analysis showed that 75 targets were associated with ferroptosis in CHIKV-induced AKI, among which seven were potential targets of oxymatrine, as shown in Figure 6A. A PPI network was constructed for the 75 ferroptosis-related targets, as illustrated in Figure 6B. Analysis using four topological algorithms identified IL1B, TNF, and IL6 as the most highly connected nodes within the network, as shown in Figure 6C.

To further clarify the role of oxymatrine in CHIKV-associated ferroptosis, the top 20 targets with the highest degree of connectivity were examined based on Venn analysis. These results are also presented in Figure 6C. Among these key targets, IFNG and TLR4 were identified as potential targets regulated by oxymatrine, suggesting a link between oxymatrine and ferroptosis-related signaling in CHIKV-induced AKI.

Molecular docking was then performed to assess the interaction between oxymatrine and ferroptosis-related proteins. As shown in Figure 6D, oxymatrine was able to bind to both human GPX4 and IL6 proteins, with Vina scores of−6.3 and−9.3, respectively, indicating stable interactions.

To validate these findings at the protein level, ELISA was used to measure inflammatory cytokines in CHIKV-infected HEK293T cells treated with oxymatrine at concentrations ranging from 1,000 μM to 500 μM. As shown in Figure 6E, oxymatrine significantly reduced the levels of IL-1β, TNF-α, and IL-6 in infected cells (*p* < 0.05), supporting its potential role in regulating ferroptosis-related inflammatory responses.

## 4 Discussion

In recent years, increasing evidence has suggested that CHIKV infection can cause multi-organ dysfunction beyond its classical musculoskeletal manifestations (Traverse et al., 2022; Cheng et al., 2025; Mercado et al., 2018). Among these complications, acute kidney injury (AKI) is considered one of the most severe and potentially life-threatening outcomes (Mercado et al., 2018). However, the molecular mechanisms underlying CHIKV-induced AKI remain poorly defined, and effective targeted therapies are still lacking. In this study, we explored the potential protective effects of oxymatrine against CHIKV-induced AKI by integrating network pharmacology, molecular docking, and *in vitro* experimental validation. Our findings indicate that oxymatrine suppresses viral replication and attenuates CHIKV-induced inflammatory responses at the cellular level.

Computational prediction analysis revealed a substantial overlap between CHIKV-related targets and AKI-associated genes. Within the protein-protein interaction (PPI) network, TNF, AKT1, IL6, IL1B, and TP53 emerged as highly connected core nodes. This distribution suggests that CHIKV-induced AKI is closely linked to dysregulated inflammatory control, altered cell survival signaling, and enhanced cellular stress responses (Voskarides and Giannopoulou, 2023; Schultheiß et al., 2022; Defois et al., 2023; Siddika et al., 2022). Further KEGG enrichment analysis consistently identified the PI3K/AKT and TNF signaling pathways as core pathways involved in CHIKV-induced AKI, as both were present across all three CHIKV-AKI subtarget groups. Notably, previous bioinformatics analyses have also highlighted the PI3K/AKT signaling pathway as a key contributor to CHIKV-associated renal injury (Cheng et al., 2025).

The PI3K/AKT signaling pathway has been reported to facilitate intracellular CHIKV replication, while sustained or abnormal activation of this pathway can disrupt cellular homeostasis and promote apoptosis and organ dysfunction (Mudaliar et al., 2021; Wikan et al., 2014). KEGG pathway enrichment analysis revealed that the intersecting targets of oxymatrine and CHIKV-induced AKI were significantly enriched in the PI3K/AKT signaling pathway, suggesting that oxymatrine may interfere with CHIKV replication through modulation of this pathway. Although no previous studies have directly investigated the effects of oxymatrine on CHIKV infection, substantial evidence indicates that oxymatrine can regulate the PI3K/AKT signaling pathway. In a mouse model of colitis, oxymatrine was shown to downregulate Th1 and Th17 cell differentiation via inhibition of the PI3K/AKT pathway, thereby markedly alleviating ulcerative colitis (Chen et al., 2017). Similarly, another study demonstrated that oxymatrine reduced p-AKT levels while upregulating PTEN expression, resulting in suppression of PI3K/AKT signaling, promotion of apoptosis, and inhibition of invasiveness in gallbladder cancer cells (Qian et al., 2018). In the present study, although key proteins involved in the PI3K/AKT pathway were not directly examined, oxymatrine was confirmed to effectively inhibit CHIKV proliferation in vitro. Notably, time-of-addition assays revealed that oxymatrine exerted antiviral effects when administered both before and after viral infection, indicating its potential to act at multiple stages of the viral life cycle.

RT-qPCR analysis further showed that oxymatrine significantly reduced the expression levels of TLR2 and TLR4 in CHIKV-infected HEK293T cells. Both genes are enriched in the PI3K/AKT signaling pathway, which further supports the involvement of this pathway in the observed effects. In addition, molecular docking analysis demonstrated strong binding between oxymatrine and AKT1, with a Vina score of−7.10. AKT1 is a central downstream kinase in the PI3K/AKT pathway and plays a major role in regulating cell survival, proliferation, metabolism, and growth (Manning and Toker, 2017). These results further indicate that oxymatrine may inhibit PI3K/AKT signaling activity.

Beyond PI3K/AKT signaling, oxymatrine also showed favorable binding affinity to SRC, NFKB1, TNF, and RELA, suggesting that its anti-CHIKV effects may involve the regulation of multiple host signaling pathways. SRC is a key non-receptor tyrosine kinase involved in the invasion and replication of various viruses, and its activity is closely associated with viral adsorption, endocytosis, and early infection processes (Ren et al., 2020; Tsai et al., 2014). NFKB1 and RELA are core transcription factors of the NF-κB signaling pathway and, together with TNF, participate in inflammatory responses and innate immune regulation (Lerebours et al., 2008; Hu et al., 2023). Previous studies have shown that CHIKV infection can markedly activate NF-κB signaling, induce excessive expression of inflammatory mediators, and aggravate host inflammatory injury (Roberts et al., 2025). The strong binding capacity of oxymatrine to these proteins suggests that it may exert anti-CHIKV effects by inhibiting abnormal activation of NF-κB and TNF-related inflammatory pathways, alleviating CHIKV-induced inflammation, and interfering with virus-associated host signaling processes. In addition to these findings, we observed that 2,000 μM oxymatrine downregulated the expression of *TIM-1*, a known CHIKV entry factor, in both infected and uninfected cells (Kirui et al., 2021). Although molecular docking indicated only moderate binding affinity between oxymatrine and TIM-1, the consistent reduction in *TIM-1* expression suggests that oxymatrine may indirectly limit CHIKV adsorption by reducing host receptor availability.

Our study also indicates that CHIKV-induced AKI may involve ferroptosis, with inflammation representing the final pathological outcome. Oxymatrine appears to inhibit this process through targets such as *IFNG* and *TLR4*. Previous analyses also demonstrated strong binding of oxymatrine to GPX4 and IL6. GPX4 is recognized as a key regulator that prevents ferroptosis by removing phospholipid peroxides and maintaining lipid oxidation homeostasis (Wang et al., 2025; Yang and Gu, 2024). In contrast, IL-6 is not a direct executor of ferroptosis but can indirectly influence this process by regulating iron metabolism and enhancing oxidative stress responses (Ferrer et al., 2023). IL-6 induces hepcidin expression, promotes intracellular iron accumulation, and increases reactive oxygen species generation through inflammation-related signaling pathways (Yang et al., 2020), thereby elevating lipid peroxidation levels (Wang et al., 2022). These findings raise the possibility that the anti-inflammatory effects of oxymatrine in CHIKV-infected HEK293T cells may be partially mediated through the modulation of ferroptosis-related mechanisms. However, it should be noted that this pathway was identified primarily through bioinformatic prediction, and the current evidence remains preliminary. Further experimental validation is required to substantiate these observations.

Several limitations of this study should be acknowledged. First, experimental validation was mainly performed in HEK293T cells. Although these cells share certain characteristics with renal epithelial cells, they do not fully represent the complexity of renal tissue *in vivo*. Second, while network pharmacology and molecular docking provided useful mechanistic insights, further studies using CHIKV-induced AKI animal models are required to confirm the renal protective effects of oxymatrine *in vivo*. Despite these limitations, our findings provide new perspectives on potential strategies for reducing renal complications associated with CHIKV infection.

## 5 Conclusion

These findings demonstrate that oxymatrine may alleviate the damage caused by the CHIKV by inhibiting viral replication, suppressing inflammatory responses, and regulating ferroptosis.

## Data Availability

The datasets presented in this study can be found in online repositories. The names of the repository/repositories and accession number(s) can be found in the article/Supplementary material.

## Supplementary material

Supplementary Material 1 Primer sequences.
