# Supplementary material for: Exploring the potential of oxymatrine in preventing CHIKV-induced acute kidney injury based on multi-dimensional computational analysis and in vitro experiments
Source: Front Microbiol. 2026 Apr 2;17:1782183. doi: 10.3389/fmicb.2026.1782183 (PMC13083131; doi:10.3389/fmicb.2026.1782183)
Supplement: Supplementary Material 1 — Primer sequences. [file Table_1.docx]

CHIKV Nsp2 (5'->3')

GGCAGTGGTCCCAGATAATTCAAG

GTACATACCCCACCTAGATCTGTCG

β-Actin (5'->3')

ATCACCATTGGCAATGAGCG

TTGAAGGTAGTTTCGTGGAT

BCL2 (5'->3')

ACGTGGACCTCATGGAGTG

TGTGTATAGCAATCCCAGGCA

BAX (5'->3')

CCCGAGAGGTCTTTTTCCGAG

CCAGCCCATGATGGTTCTGAT

CASP3 (5'->3')

CATGGAAGCGAATCAATGGACT

CTGTACCAGACCGAGATGTCA

CTSB (5′→3′)

GAGCTGGTCAACTATGTCAACA

GCTCATGTCCACGTTGTAGAAGT

TLR4 (5′→3′)

AGACCTGTCCCTGAACCCTAT

AGACCTGTCCCTGAACCCTAT

MMP9 (5′→3′)

TGTACCGCTATGGTTACACTCG

GGCAGGGACAGTTGCTTCT

TLR2 (5′→3′)
ATCCTCCAATCAGGCTTCTCT
GGACAGGTCAAGGCTTTTTACA

ACTG1 (5′→3′)
CCGAGCCGTGTTTCCTTCC
GCCATGCTCAATGGGGTACT

FHL1 (5′→3′)
TGCTGCCTGAAATGCTTTGAC
GCCAGAAGCGGTTCTTATAGTG

TIM-1 (5′→3′)
AACTGTCTCTACCTTGTTCCTCC
GTTCTCTCCTTATTGCTCCCTG

COL1A2 (5′→3′)
GTGGCAGTGATGGAAGTGTG
AGGACCAGCGTTACCAACG

PTPN2 (5′→3′)
GCAGTGAGAGCATTCTACGGA
TGACACAAACCCCATCTTAGTGA

IFITM3 (5′→3′)
GACCATTCTGCTCATCGTCATC
AGCCAGACCCTCCCAATGTT
