# Supplementary figures and images for: Exploring the potential of oxymatrine in preventing CHIKV-induced acute kidney injury based on multi-dimensional computational analysis and in vitro experiments
Source: Front Microbiol. 2026 Apr 2;17:1782183. doi: 10.3389/fmicb.2026.1782183 (PMC13083131; doi:10.3389/fmicb.2026.1782183)

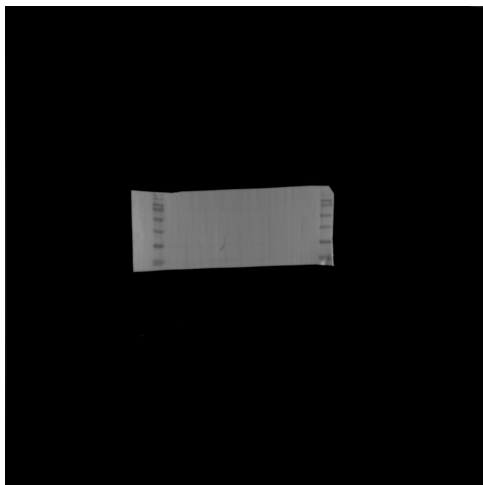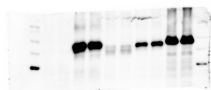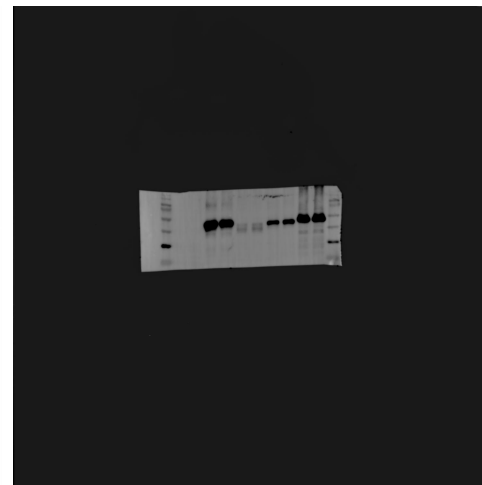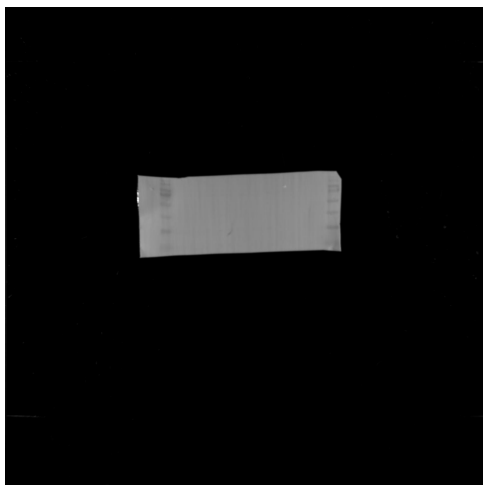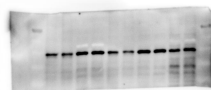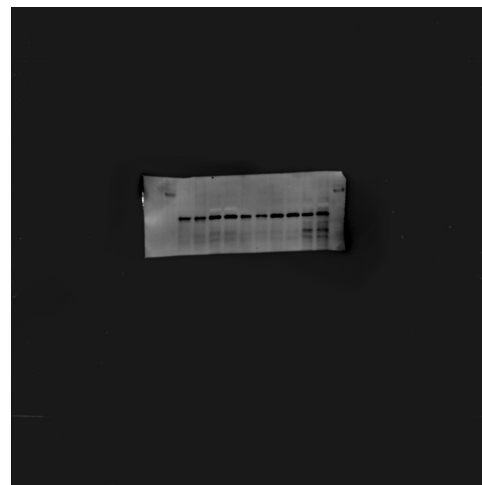

Supplement: Supplementary file 2 [file Data_Sheet_1.pdf]
